# The Disease-Free Interval Between Resection of Primary Colorectal Malignancy and the Detection of Hepatic Metastases Predicts Disease Recurrence But Not Overall Survival

**DOI:** 10.1245/s10434-019-07481-x

**Published:** 2019-05-30

**Authors:** Diederik J. Höppener, Pieter M. H. Nierop, Martinus J. van Amerongen, Pim B. Olthof, Boris Galjart, Thomas M. van Gulik, Johannes H. W. de Wilt, Dirk J. Grünhagen, Nuh N. Rahbari, Cornelis Verhoef

**Affiliations:** 1 Department of Surgical Oncology and Gastrointestinal Surgery, Erasmus MC Cancer Institute, Rotterdam, The Netherlands; 2 Department of Radiology, Radboud University Medical Center, Radboud University Nijmegen, Nijmegen, The Netherlands; 3 Department of Surgery, Amsterdam UMC, University of Amsterdam, Amsterdam, The Netherlands; 4 Department of Surgery, Reinier de Graaf Gasthuis, Delft, The Netherlands; 5 Department of Surgery, Radboud University Medical Center, Radboud University Nijmegen, Nijmegen, The Netherlands; 6 Department of Surgery, Mannheim University Medical Center, University of Heidelberg, Mannheim, Germany

## Abstract

**Introduction:**

The disease-free interval (DFI) between resection of primary colorectal cancer (CRC) and diagnosis of liver metastases is considered an important prognostic indicator; however, recent analyses in metastatic CRC found limited evidence to support this notion.

**Objective:**

The current study aims to determine the prognostic value of the DFI in patients with resectable colorectal liver metastases (CRLM).

**Methods:**

Patients undergoing first surgical treatment of CRLM at three academic centers in The Netherlands were eligible for inclusion. The DFI was defined as the time between resection of CRC and detection of CRLM. Baseline characteristics and Kaplan–Meier survival estimates were stratified by DFI. Cox regression analyses were performed for overall (OS) and disease-free survival (DFS), with the DFI entered as a continuous measure using a restricted cubic spline function with three knots.

**Results:**

In total, 1374 patients were included. Patients with a shorter DFI more often had lymph node involvement of the primary, more frequently received neoadjuvant chemotherapy for CRLM, and had higher number of CRLM at diagnosis. The DFI significantly contributed to DFS prediction (*p *=0.002), but not for predicting OS (*p *=0.169). Point estimates of the hazard ratio (95% confidence interval) for a DFI of 0 versus 12 months and 0 versus 24 months were 1.284 (1.114–1.480) and 1.444 (1.180–1.766), respectively, for DFS, and 1.111 (0.928–1.330) and 1.202 (0.933–1.550), respectively, for OS.

**Conclusion:**

The DFI is of prognostic value for predicting disease recurrence following surgical treatment of CRLM, but not for predicting OS outcomes.

**Electronic supplementary material:**

The online version of this article (10.1245/s10434-019-07481-x) contains supplementary material, which is available to authorized users.

The liver remains the most frequent metastatic site for patients with colorectal cancer (CRC), with approximately 30–40% of all patients diagnosed with CRC developing colorectal liver metastases (CRLM) over the course of their disease.^1^^–^4 Curatively intended treatment of CRLM has increasingly been performed for more advanced disease, with surgical resection being the mainstay of treatment.^5^ This is in part due to increasing local and systemic treatment options (e.g. ablative therapies and preoperative chemotherapy), and more extensive (multistaged) surgical strategies (e.g. two-staged hepatectomies, Associating Liver Partition and Portal vein ligation for Staged hepatectomy [ALPPS], and the liver-first approach).^3^^,^^6^^–^13 Despite these advancements over time, there still remains room for improvement, with most studies reporting 5-year overall survival (OS) rates of 40–60%.^10^^,^^14^^,^^15^

Multiple clinical risk scores have been proposed to predict survival outcomes after surgical treatment for CRLM.^16^^–^20 The disease-free interval (DFI), defined as the time between resection of the primary malignancy and the diagnosis of CRLM, is considered a predictor of tumor biology and prognosis. Recent analysis of the German population-based case–control DACHS study found no noticeable predictive value of the time to metastases on survival outcomes in CRC patients.^21^ The analysis was performed in 1027 patients diagnosed with CRC who developed metastatic disease or had synchronous metastatic disease at the time of CRC diagnosis. The authors did not distinguish between type of metastatic disease or whether patients were eligible for curative local therapy. Therefore, the aim of the present study was to assess the prognostic value of the DFI, specifically in patients with CRLM eligible for surgical treatment.

## Methods

The current study was approved by the Medical Ethics Committee of the Erasmus Medical Center (MEC-2018-1743).

### Patient Selection

All consecutive patients undergoing surgical treatment for CRLM at the Erasmus MC Cancer Institute between January 2000 and December 2016, at the Radboud University Medical Center between July 2000 and June 2018, and at the Academic Medical Center of the Amsterdam UMC between November 2006 and September 2015 were eligible for inclusion. Patients with extrahepatic disease at the time of surgery, as well as patients undergoing treatment for recurrent CRLM, were excluded. In addition, the DFI had to be known and patients had to be considered tumor-free following surgery for CRLM, or following resection of the primary malignancy in case of a liver-first approach.

### Data Collection and Definitions

Patient data and clinicopathological characteristics of the primary malignancy and CRLM were extracted from prospectively maintained databases. The DFI was defined as the time interval (months) between resection of the primary colorectal malignancy and detection of CRLM. The date of detection of CRLM was defined as the date of medical imaging on which metastasis was first diagnosed. The DFI of patients in whom CRLM were diagnosed prior to or simultaneously with the primary CRC was considered zero. OS was defined as the interval between surgery for CRLM and death, while disease-free survival (DFS) was defined as the interval between surgical treatment of hepatic metastasis and date of diagnosis of disease recurrence or death. In case of absent recurrent disease or death, patients were censored at the date of last follow-up.

### Treatment and Follow-Up of Patients with Colorectal Liver Metastases

All three participating centers are tertiary referral centers for liver surgery. A multidisciplinary tumor board evaluates all patients referred for treatment of CRLM, to establish optimal treatment strategy. In The Netherlands, perioperative chemotherapy for CRLM is not considered standard of care. In case of marginally resectable disease, preoperative chemotherapy (oxaliplatin- or irinotecan-based) is utilized in an effort to increase resectability and optimize surgical treatment options. Follow-up of patients after surgical treatment of CRLM is performed for up to 5 years according to Dutch guidelines. Serial carcinoembryonic antigen (CEA) measurements are performed on a 3-monthly basis, while medical imaging by computed tomography (CT) or magnetic resonance imaging (MRI) is usually performed semi-annually for the first 2 years and annually thereafter. The optimal treatment strategy for recurrent disease is again determined by a multidisciplinary tumor board. In case of liver-limited disease eligible for local treatment with sufficient remnant liver volume, salvage local therapy is generally attempted. In case of concurrent oligometastasic extrahepatic disease, salvage local treatment is still deemed feasible. In case of extrahepatic disease in more than one organ, salvage local treatment is usually not pursued, but patients are treated with palliative systemic therapy. Herein (repeat hepatic) resections, ablations, and stereotactic radiotherapy are considered local (i.e. curative intent) therapies. Yttrium-90 (Y90) radio embolization is not considered as curative intent treatment and is only performed in case of progressive liver-limited disease after first- and second-line chemotherapy regimens. In general, Y90 radio embolization is infrequently performed in The Netherlands.

### Statistical Analysis

Categorical data are reported as counts with corresponding percentages, and continuous data are reported as median with corresponding interquartile range (IQR). Baseline categorical and numerical variables were compared using the Chi square and nonparametric Mann–Whitney *U* or Kruskal–Wallis tests (depending on the number of strata), respectively. Kaplan–Meier survival estimates were generated using the log-rank test to compare across strata, and median follow-up for survivors was determined using the reverse Kaplan–Meier method. Baseline comparison and Kaplan–Meier OS and DFS estimates were stratified by DFI (0–1, 2–12, 13–24, and > 24 months), and Cox multivariable regression analysis for OS and DFS was performed. No stepwise selection of predictors was applied. All variables considered for multivariable regression analysis comprised of known clinical risk and treatment-related factors. With regard to the resection margin in regression analysis, patients treated solely with ablative therapy were considered R0. To allow for possible non-linear relationships, all numerical and ordinal variables including the DFI were entered continuously using restricted cubic spline functions with three knots. Results were reported using the Wald statistic for improvement of model fit for individual variables and their corresponding *p* value. The relative hazard by DFI (months) on OS and DFS was graphically displayed using partial effect plots. Due to the use of non-linear terms (i.e. restricted cubic spline functions), no singular hazard ratio (HR) with corresponding 95% confidence interval (CI) for the DFI on OS and DFS can be given. Therefore, point estimates of the HR and 95% CI for OS and DFS were calculated for a DFI of 0 versus 12 months, 0 versus 24 months, 0 versus 36 months, and 0 versus 48 months, respectively. To assess a possible effect of DFI on eligibility for salvage local treatment, multivariable logistic regression analysis on salvageable recurrence was performed using restricted cubic spline functions with three knots for all numerical or ordinal predictors. A partial effect plot was used to graphically display the odds for salvage local treatment by DFI (months). All statistical analyses were performed using R version 3.5.1 (http://www.r-project.org), and the R-package ‘rms’ was used to perform regression analysis with restricted cubic spline functions.

## Results

Between January 2000 and December 2016, a total of 840 eligible patients who underwent surgical treatment of CRLM at the Erasmus MC Cancer Institute were identified. Median follow-up for survivors was 67 months (IQR 37–111). During follow-up, disease recurrence was diagnosed in 568 (68%) patients and 416 (50%) patients died. At the Radboud University Medical Center, 385 eligible patients were operated on between July 2000 and June 2018. Median follow-up for survivors was 32 months (IQR 14–57). Disease recurrence was detected in 229 (59%) patients and 96 (25%) patients died. From November 2006 to September 2015, a total of 149 eligible patients underwent surgery at the Academic Medical Center. Median follow-up for survivors was 54 months (IQR 29–78). Disease recurrence was diagnosed in 81 (54%) patients and 66 (44%) patients died during follow-up. In the combined cohort of 1374 patients, the median follow-up for survivors was 54 months (IQR 26–90).

Baseline characteristics stratified by DFI are reported in Table 1. In general, patients with a shorter DFI were younger, more often had a higher T stage and rate of lymph node involvement of the primary tumor, more frequently received neoadjuvant chemotherapy for CRLM, and had more CRLM at diagnosis. Patients with a longer DFI more often received adjuvant chemotherapy following CRC resection and were found to have larger CRLM.

**Table 1:** Baseline characteristics stratified by disease-free interval

	Disease-free interval, months				*p* value
	0–1 [*n* = 682 (%^a^)]	2–12 [*n* = 297 (%^a^)]	13–24 [*n* = 211 (%^a^)]	> 24 [*n* = 184 (%^a^)]	
Age at resection CRLM (median [IQR])	64.0 [56.0–70.0]	65.0 [60.0–72.0]	67.0 [60.0–74.0]	67.5 [60.8–73.0]	< 0.001*
*Sex*					
Female	257 (38)	106 (36)	70 (33)	57 (31)	0.315
Male	425 (62)	191 (64)	141 (67)	127 (69)	
*ASA classification*					
I–II	601 (89)	249 (85)	182 (87)	159 (89)	0.434
> II	76 (11)	43 (15)	28 (13)	20 (11)	
*Missing*	5 (1)	5 (2)	1 (0)	5 (3)	
*Primary tumor location*					
Left-sided	290 (43)	101 (35)	91 (44)	90 (50)	0.044*
Rectal	227 (34)	113 (39)	75 (36)	58 (32)	
Right-sided	152 (23)	76 (26)	43 (21)	31 (17)	
*Missing*	13 (2)	7 (2)	2 (1)	5 (3)	
*T stage*					
pT 0–2	106 (16)	61 (21)	50 (24)	43 (24)	0.011*
pT 3–4	565 (84)	231 (79)	158 (76)	138 (76)	
*Missing*	11 (2)	5 (2)	3 (1)	3 (2)	
*N stage*					
*N*0	212 (32)	134 (46)	94 (45)	99 (55)	< 0.001*
*N *+	457 (68)	160 (54)	113 (55)	82 (45)	
*Missing*	13 (2)	3 (1)	4 (2)	3 (2)	
*Adjuvant CTx for CRC*					
No	568 (96)	175 (78)	108 (65)	105 (70)	< 0.001*
Yes	22 (4)	50 (22)	57 (35)	46 (30)	
*Missing*	92 (13)	72 (24)	46 (22)	33 (18)	
*Neoadjuvant CTx for CRLM*					
No	243 (36)	211 (71)	169 (80)	140 (76)	< 0.001*
Yes	439 (64)	86 (29)	42 (20)	44 (24)	
Number of CRLM (median [IQR])	2.0 [1.0–4.0]	2.0 [1.0–3.0]	1.0 [1.0–2.0]	1.0 [1.0–2.0]	< 0.001*
Diameter of the largest CRLM (median [IQR])	2.8 [1.9–4.1]	2.8 [2.0–4.0]	3.0 [2.0–4.1]	4.0 [2.6–5.5]	< 0.001*
Preoperative CEA (median [IQR])	10.8 [3.6–44.3]	12.0 [3.9–34.8]	11.0 [4.3–27.8]	13.5 [5.2–33.4]	0.798
*Resection margin CRLM*					
*R*0	581 (86)	261 (88)	178 (86)	155 (85)	0.730
*R*1	91 (14)	34 (12)	29 (14)	27 (15)	
*Missing*	10 (1)	2 (1)	4 (2)	2 (1)	
*Clinical risk score*					
Low	269 (42)	178 (63)	187 (92)	171 (95)	< 0.001*
High	367 (58)	105 (37)	17 (8)	9 (5)	
*Missing*	46 (7)	14 (5)	7 (3)	4 (2)	
*Hemihepatectomy*					
No	494 (73)	231 (78)	157 (74)	127 (69)	0.169
Yes	186 (27)	66 (22)	54 (26)	57 (31)	
*Missing*	2 (0)	0 (0)	0 (0)	0 (0)	
*Postoperative complications*					
None or grade 1–2	592 (87)	254 (86)	185 (89)	165 (90)	0.607
Grade 3 or higher	88 (13)	41 (14)	23 (11)	19 (10)	
*Missing*	2 (0)	2 (1)	3 (1)	0 (0)	
*Postoperative mortality*					
No	672 (99)	289 (98)	207 (100)	181 (98)	0.362
Yes	9 (1)	7 (2)	1 (0)	3 (2)	
*Missing*	1 (0)	1 (0)	3 (1)	0 (0)	

*CRLM* colorectal liver metastases, *IQR* interquartile range, *ASA* American Society of Anesthesiologists, *CTx* chemotherapy, *CRC* colorectal cancer, *CEA* carcinoembryonic antigen

^a^Percentages are expressed as proportions across each stratum (i.e. excluding ‘missing’). Percentages for ‘missing’ are expressed as a proportion of missing values within each stratum

*α < 0.05

**Fig. 1:**
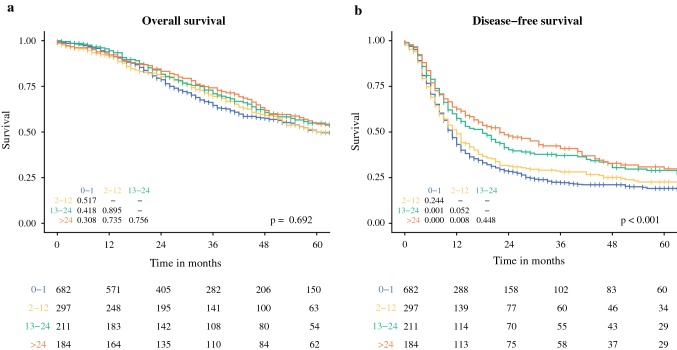
Kaplan–Meier survival curves for (**a**) overall survival and (**b**) disease-free survival stratified by the disease-free interval (months). The overall *p* value is displayed in the bottom right-hand corner of each graph; *p* values of the pairwise comparison of individual strata are reported in the left-hand corner; and the numbers at risk per stratum are reported in the table below the graphs

Kaplan–Meier survival curves for OS and DFS from resection of CRLM stratified by DFI are shown in Fig. 1. No overall or pairwise differences were found for OS (overall *p *=0.692). For DFS, an overall significant difference between groups was found (*p *<0.001), with individual comparisons showing a significant impaired DFS for a DFI of 0–1 compared with 13–24 and > 24 months (*p *=0.001 and *p *<0.001), and for a DFI of 2–12 compared with > 24 months (*p *=0.008).

**Fig. 2:**
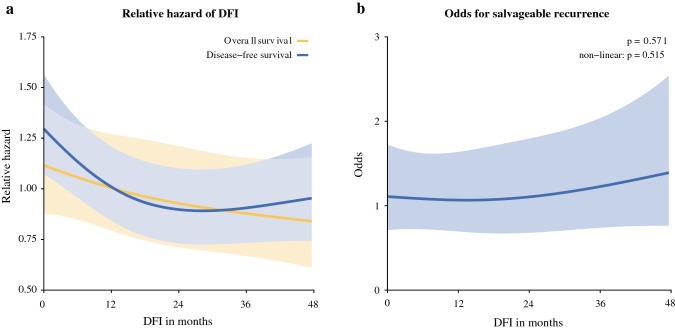
(**a**) Partial effect plot of the relative hazard, with corresponding 95% confidence band, of the DFI (months) on disease-free and overall survival. (**b**) Partial effect plot of the odds for salvageable recurrence, with corresponding 95% confidence band, for the DFI (months). *DFI* disease-free interval

Results of multivariable regression models on OS and DFS are reported in Table 2. No significant effect was found for the DFI on OS (*p *=0.169). In multivariable analysis for DFS, the DFI proved to be a significant predictor (*p *=0.002). Figure 2a shows the partial effect plots of the relative hazard, with corresponding 95% confidence band of the DFI (months). The relative hazard for DFI on OS appears constant, while the relative hazard for developing a recurrence decreases as the DFI increases. This decrease is seen for a DFI up to 24 months. These results are reflected in the point estimates of the HR and 95% CI for OS and DFS comparing a DFI of 0 versus 12 months, 0 versus 24 months, 0 versus 36 months, and 0 versus 48 months (Table 3).

**Table 2:** Wald tests for improvement of multivariable Cox proportional hazard models fit for overall and disease-free survival

	Overall survival		Disease-free survival	
	Wald statistic	*p* value	Wald statistic	*p* value
Age at resection of CRLM, years^a^	26.528	< 0.001*	12.827	0.002*
Non-linear terms	1.441	0.230	1.62	0.203
Sex (male and female)	0.502	0.479	0.164	0.686
ASA classification (I–II and > II)	1.729	0.189	0.644	0.422
Primary tumor location (left-sided, right-sided, and rectal)	7.064	0.029*	4.705	0.095
*T* stage (pT0–2 and pT3–4)	0.074	0.786	0.407	0.524
*N* stage (*N*0 and *N* +)	16.345	< 0.001*	25.479	< 0.001*
Disease-free interval, months^a^	3.559	0.169	12.873	0.002*
Non-linear terms	0.168	0.682	7.315	0.007*
Number of CRLM^a^	13.105	0.001*	33.296	< 0.001*
Non-linear terms	3.571	0.059	10.667	0.001*
Diameter of the largest CRLM, cm^a^	17.171	< 0.001*	6.267	0.044*
Non-linear terms	3.817	0.051	0.738	0.390
Preoperative CEA, µg/L	5.63	0.060	8.597	0.014*
Non-linear terms	5.427	0.020*	7.561	0.006*
Neoadjuvant chemotherapy (yes and no)	2.797	0.094	7.87	0.005*
Resection margin (*R*0 and *R*1)	6.542	0.011*	19.174	< 0.001*

*CRLM* colorectal liver metastases, *ASA* American Society of Anesthesiologists, *CEA* carcinoembryonic antigen

^a^Entered as a continuous measure using a restricted cubic spline function with three knots

*α < 0.05

**Table 3:** Point estimates for the DFI of multivariable Cox regression models for overall and disease-free survival

Disease-free interval	Overall survival [HR (95% CI)]	Disease-free survival [HR (95% CI)]
0 versus 12 months	1.111 (0.928–1.330)	1.284 (1.114–1.480)
0 versus 24 months	1.202 (0.933–1.550)	1.444 (1.180–1.766)
0 versus 36 months	1.270 (0.980–1.647)	1.431 (1.164–1.758)
0 versus 48 months	1.330 (0.984–1.799)	1.356 (1.070–1.720)

*DFI* disease-free interval, *HR* hazard ratio, *CI* confidence interval

Results of the multivariable analysis on the odds for salvageable recurrence are reported in electronic supplementary Table 1. Significant predictors found for salvageable recurrence were location of the primary tumor (*p *<0.001), CRC nodal status (*p *=0.011), and preoperative CEA levels (*p *=0.028). The DFI did not significantly contribute to the multivariable model prediction (*p *=0.571). The partial effect plot in Fig. 2b displays the odds, with corresponding 95% confidence band, for a salvageable recurrence by the DFI (months). The odds for a salvageable recurrence appear constant over the DFI.

## Discussion

The results of the current study show that patients with a shorter DFI more often had high clinical risk characteristics, such as nodal positive primaries, and more metastases at the time of diagnosis. Despite the seemingly higher clinical risk at baseline, the DFI was only found to be of prognostic value for predicting disease recurrence, but not for predicting OS.

This implies that patients with a shorter DFI develop earlier disease recurrence that is not associated with impaired OS. While this might be due to applied treatment for recurrent disease, the current study showed that eligibility for salvage local treatment was independent of the DFI. These results appear contradictory to the general hypothesis that time to cancer recurrence determines prognosis. A possible explanation for this might be that it matters less when disease recurrence occurs (or when it is diagnosed), but more whether it is eligible for salvage local treatment that is most important for OS outcomes. The current study found no difference in eligibility for salvage local treatment between patients with a short or long DFI, which could explain why OS was independent of the DFI.

While this might seem contradictory to common established cancer theories, several studies have been performed that support this hypothesis. The EORTC 40983 trial randomized 364 patients between perioperative FOLFOX4 chemotherapy with surgery versus surgery alone for resectable CRLM.^22^ The addition of chemotherapy prolonged progression-free survival in patients eligible for surgery (*p *=0.035); however, long-term results showed that the percentage of patients who eventually developed cancer progression was equal: 130 (71%) in the surgery-only group versus 124 (68%) in the perioperative chemotherapy group. No difference in long-term OS was observed (*p *=0.303), and no difference was found in the amount of patients eligible for repeat resection (40 vs. 46%) or radiotherapy (2 vs. 9%). This study therefore demonstrates that perioperative chemotherapy seems to delay, but not prevent, disease progression. The number of patients who eventually develop progressive disease and the eligibility for subsequent salvage treatment remains equal, therefore no difference in OS is observed.

Similarly, with the addition of the recent COLOFOL trial,^23^ 15 randomized controlled trials evaluated a more intensive versus a less intensive postoperative surveillance after surgery for stage I–III CRC.^24^ Pooled meta-analysis found that while intensive surveillance anticipates disease recurrence, this does not translate into a cancer-specific survival benefit.^25^ The three largest and most recent trials had a combined sample size of 4939 patients,^23^^,^^26^^,^^27^ with all three trials reporting no difference in OS outcomes. Two of these trials reported on eligibility for salvage local treatment, which was equal between the intensive and less intensive surveillance groups.^26^^,^^27^ Intensive surveillance allows us to detect disease recurrence earlier, but this does not translate into a larger amount of people eligible for local treatment, and thus no OS difference is achieved.

Repeat hepatic resection for recurrent CRLM has proven feasible, and reported long-term survival outcomes are comparable with those after first surgical treatment.^28^^–^32 It would therefore be interesting to assess the effect of the DFI between hepatic resections on survival outcomes following second liver resection for CRLM. The interval between CRLM surgery and the date of hepatic recurrence has proven to be of prognostic value in patients undergoing repeat hepatic resection.^29^ Patients with an interval of < 6 months exhibited impaired OS. While some studies reported similar results,^31^ others did not.^30^^,^^32^ In addition, retrospective analysis of such a cohort is highly subject to selection bias. Clinical decision making is largely influenced by the ‘known clinical risk factors’, of which this interval is generally considered to be one. Therefore, interpretation of the prognostic value of the DFI between hepatic resections should be done with caution until reliable prospective data become available.

The results of the current study are insufficient to denounce any prognostic value of the DFI on OS outcomes after surgical treatment of CRLM. This study is limited in its retrospective design. No data were available for the adherence to follow-up scan protocols, which is an important determinant of the DFS. In addition, lead time bias remains an important potential confounder. It is possible that patients with a shorter DFI are more often ineligible for surgical treatment, or are considered unresectable during surgery. Nevertheless, in those patients who actually undergo surgical treatment of CRLM, the DFI does not seem a strong prognostic indicator for OS. These findings are in line with the results reported by Rahbari et al.^21^ It is therefore reasonable to assume that the prognostic value of the DFI on OS following curative surgery for CRLM is more restricted than previously thought. Since the evolution of surgical treatment for CRLM, the factors on which clinical predictions are made have remained largely unchanged. Studies like this emphasize the limitations of predicting OS outcomes in (colorectal) cancer patients based solely on clinical predictors. Efforts should be made to improve predictions of survival outcomes, possibly by relying more on prediction based on underlying biological factors. In (stage IV) CRC, there is increasing evidence that prognosis and treatment effect can be predicted by biological variables, such as preoperative skeletal muscle mass,^33^ the histopathological growth pattern of CRLM,^34^^–^40 mutational status,^41^^–^44 and the quantification of immune infiltration at the tumor microenvironment,^45^^–^47 among others.

## Conclusion

Following surgical treatment of CRLM, the DFI is of prognostic value for predicting disease recurrence, but not for predicting OS outcomes. In an effort to improve clinical prediction and decision making in patients with CRLM, prognostic models need to be based more on underlying biology rather than relying solely on clinical factors such as the DFI.

## Electronic supplementary material

Below is the link to the electronic supplementary material.

Supplementary material 1 (DOCX 15 kb)
