# Supplementary material for: The Disease-Free Interval Between Resection of Primary Colorectal Malignancy and the Detection of Hepatic Metastases Predicts Disease Recurrence But Not Overall Survival
Source: Ann Surg Oncol. 2019 May 30;26(9):2812–20. doi: 10.1245/s10434-019-07481-x (PMC6682566; doi:10.1245/s10434-019-07481-x)
Supplement: Supplementary file 1 — Supplementary material 1 (DOCX 15 kb) [file 10434_2019_7481_MOESM1_ESM.docx]

| **Supplementary table 1.** Wald tests for improvement of multivariable logistic regression model fit for salvageable recurrence | | | |
| --- | --- | --- | --- |
|  |  | Wald statistic | p-value |
| Age at resection CRLM - *years* ^a^ | | 2.339 | 0.311 |
|  | Non-linear terms | 0.658 | 0.417 |
| Gender *- male & female* | | 0.907 | 0.341 |
| ASA classification - *I-II & >II* | | 0.062 | 0.803 |
| Primary tumour location - l*eft-sided, right-sided & rectal* | | 20.91 | <0.001* |
| T-stage - *pT0-2 & pT3-4* | | 0.902 | 0.342 |
| N-stage - *N0 & N+* | | 6.552 | 0.010* |
| Disease-free interval - *months* ^a^ | | 1.122 | 0.571 |
|  | Non-linear terms | 0.424 | 0.515 |
| Number of CRLM ^a^ | | 4.728 | 0.094 |
|  | Non-linear terms | 1.551 | 0.213 |
| Diameter of largest CRLM - *cm* ^a^ | | 5.769 | 0.056 |
|  | Non-linear terms | 0.135 | 0.713 |
| Preoperative CEA - *µg/L* ^a^ | | 7.139 | 0.028* |
|  | Non-linear terms | 2.795 | 0.095 |
| Neo-adjuvant chemotherapy - yes & *no* | | 3.378 | 0.066 |
| Resection margin - *R0 & R1* | | 0.016 | 0.899 |
| Abbreviations: *CRLM: colorectal liver metastasis, CEA: carcinoembryonic antigen* ^a^ *Entered as continuous measure using a restricted cubic spline function with 3 knots ** α *< 0.05* | | | |
|  |  |  |  |
|  |  |  |  |
